# The role of PIP5K1α/pAKT and targeted inhibition of growth of subtypes of breast cancer using PIP5K1α inhibitor

**DOI:** 10.1038/s41388-018-0438-2

**Published:** 2018-08-13

**Authors:** Martuza Sarwar, Azharuddin Sajid Syed Khaja, Mohammed Aleskandarany, Richard Karlsson, Maryam Althobiti, Niels Ødum, Nigel P. Mongan, Nisthman Dizeyi, Heather Johnson, Andrew R. Green, Ian O. Ellis, Emad A. Rakha, Jenny L Persson

**Affiliations:** 1 Division of Experimental Cancer Research, Department of Translational Medicine, Lund University, Clinical Research Centre, Malmö, Sweden; 2 Division of Basal Tumor Biology, Department of Molecular Biology, Umeå University, Umeå, Sweden; 3 Academic Pathology, Division of Cancer and Stem Cells, School of Medicine, University of Nottingham, Nottingham, UK; 4 Department of Immunology and Microbiology, University of Copenhagen, Copenhagen, Denmark; 5 Faculty of Medicine and Health Sciences, School of Veterinary Medicine and Science, University of Nottingham, Nottingham, United Kingdom; 6 Department of Pharmacology, Weill Cornell Medicine, New York, NY 10065 USA; 7 Division of reproductive research, Department of Translational Medicine, Lund University, Clinical Research Centre, Malmö, Sweden; 8 Department of Bio-Diagnosis, Beijing Institute of Basic Medical Sciences, Beijing, China

**Keywords:** Breast cancer, Predictive markers

## Abstract

Despite recent improvement in adjuvant therapies, triple-negative, and ER^+^ subtypes of breast cancer (BC) with metastatic potentials remain the leading cause of BC-related deaths. We investigated the role of phosphatidylinositol-4-phosphate 5-kinase alpha (PIP5Kα), a key upstream factor of PI3K/AKT, and the therapeutic effect of PIP5Kα inhibitor on subtypes of BC. The clinical importance of PIP5K1α and its association with survivals were analyzed using three BC cohorts from Nottingham (*n* = 913), KM plotter (*n* = 112) and TCGA (*n* = 817). Targeted overexpression or knockdown of PIP5K1α were introduced into BC cell lines. The effects of PIP5K1α and its inhibitor on growth and invasion of BC were confirmed by using in vitro assays including proliferation, migration, apoptosis and luciferase reporter assays and in vivo xenograft mouse models. All statistical tests were two-sided. PIP5K1α was associated with poor patient outcome in triple-negative BC (for PIP5K1α protein, *p* = 0.011 and for mRNA expression, *p* = 0.028, log-rank test). 29% of triple-negative BC had *PIP5K1A* gene amplification. Elevated level of PIP5K1α increased expression of pSer-473 AKT (*p* < 0.001) and invasiveness of triple-negative MDA-MB-231 cells (*p* < 0.001). Conversely, inhibition of PIP5K1α using its inhibitor ISA-2011B, or via knockdown suppressed growth and invasiveness of MDA-MB-231 xenografts (mean vehicle-treated controls = 2160 mm^3^, and mean ISA-2011B-treated = 600 mm^3^, *p* < 0.001). ISA-2011B-treatment reduced expression of pSer-473 AKT (*p* < 0.001) and its downstream effectors including cyclin D1, VEGF and its receptors, VEGFR1 and VEGFR2 (*p* < 0.001) in xenograft tumors. In ER^+^ cancer cells, PIP5K1α acted on pSer-473 AKT, and was in complexes with VEGFR2, serving as co-factor of ER-alpha to regulate activities of target genes including cyclin D1 and CDK1. Our study suggests that our developed PIP5K1α inhibitor has a great potential on refining targeted therapeutics for treatment of triple-negative and ER^+^ BC with abnormal PI3K/AKT pathways.

## Introduction

Breast cancer is a disease of heterogeneity at clinical, morphological, molecular, and genomic levels [1]. Patients with luminal breast cancers that are hormone receptor positive (estrogen receptor (ER^+^) and progesterone receptor (PR^+^)), may suffer cancer recurrence, which often metastasize to bone but respond well to endocrine therapies [2, 3]. Triple-negative or basal-like breast cancers, which are defined as breast cancer without expression of ER, PR and human epidermal growth factor receptor 2 (HER2), are found to be associated with shorter time to recurrence and higher metastatic potentials [4–6]. They do not respond to endocrine therapies, thereby represent a major clinical challenge [7, 8].

A complex network of signaling pathways may contribute to distant metastasis, which is the major cause of death of breast cancer [1, 9, 10]. The pathways often converge at the phosphatidylinositol 3-kinase (PI3K)/AKT pathway, which is a central player that controls cell cycle, survival, metabolism, motility, and genomic instability [11]. Indeed, PIK3CA mutations or PTEN loss resulting in constitutively activation of PI3K/AKT are frequently observed in ER^+^ luminal subtype and in triple-negative, basal-like breast cancers as well [7]. Thus, there is an intense interest in identifying novel approaches to inhibit PI3K/AKT.

Phosphatidylinositol 4-phosphate 5-kinase alpha (PIP5K1α) is responsible for the synthesis of PtdIns-4,5-P_2_ (PIP2), which is in turn used by PI3K to produce PtdIns-3,4,5-P_3_ (PIP3). PIP3 plays an important role in activation of AKT [12, 13], therefore, PIP5K1α acts on upstream of the PI3K/AKT/PTEN pathways and can regulate cell proliferation, apoptosis, and migration. Overexpression of PIP5K1α has been detected in MDA-MB-231 cell line, showing its involvement in breast cancer [14]. Previously we reported that a selective PIP5K1α inhibitor, ISA-2011B, blocked the PI3K/AKT pathway by decreasing AKT phosphorylation at Serine 473 (pAKT S473), leading to reduced growth of aggressive prostate tumors in a xenograft mouse model [15, 16]. Therefore, it is necessary to investigate the therapeutic potential of ISA-2011B on inhibiting the PI3K/AKT pathway in breast cancer as a targeted and possibly more effective therapy for patients with activated PI3K/AKT [17–21].

In this paper, we studied the involvement of PIP5K1α with the PI3K/AKT pathway in triple-negative and luminal ER^+^ breast cancers. We aimed at exploring the utility of ISA-2011B for treatment of metastatic breast cancer models by blocking constitutively activated PI3K/AKT pathway in preclinical settings. Our results suggest a role of PIP5K1α and ISA–2011B in targeted treatment of metastatic breast cancer.

## Results

### Expression of PIP5K1α in specimens of primary breast cancer patients

**Fig. 1:**
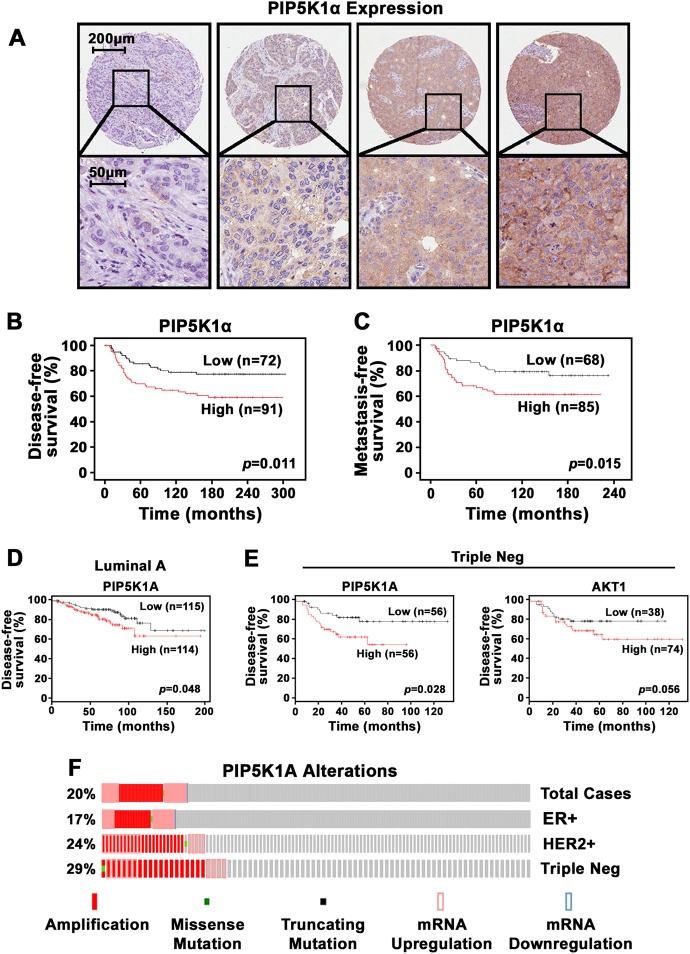
Expression of PIP5K1α protein and mRNA correlates with patient outcome in in triple-negative subtypes of breast cancer. **a** Immunohistochemical analysis of TMAs containing primary tumor tissues from 913 breast cancer patients using antibody against PIP5K1α. Representative microphotographs showing PIP5K1α expression and subcellular localization in various types of breast cancer tissues. **b**, **c** Kaplan–Meier survival analysis based on disease-free (**b**) or distant metastasis-free (**c**) shows the difference between patients with low or high expression of PIP5K1α in a triple-negative breast cancer patient cohort. **d** Kaplan–Meier survival analysis shows the differences between patients with high or low *PIP5K1A* in Lumina A breast cancer patient cohort. **e** Kaplan-Meier survival analysis shows the differences between patients with high or low *PIP5K1A/AKT1* mRNA levels in a triple-negative breast cancer patient cohort. Differences in disease-free or metastasis-free survivals between two groups were calculated using the log-rank test. *P* values are indicated. **f** Alterations in *PIP5K1A* in 20% (*n* = 164) of unselected cases of breast cancer (*n* = 817 cases), and in 17% (*n* = 102) of ER^+^ subtype (*n* = 594), and in 24% (*n* = 29) of HER-2 positive subtype (*n* = 120) and in 29% (*n* = 24) of triple-negative subtypes (*n* = 82)

We examined PIP5K1α expression using tissue microarrays (TMAs) consisting of molecular subtypes including luminal subtypes and basal-like classes [22]. Expression of PIP5K1α protein was detected in breast cancer tissues of various subtypes (Fig. 1a). In the luminal subtype, higher level of PIP5K1α was shown to be associated with high-grade cancers and cancers with poor prognosis, respectively (*p* = 0.031 and *p* = 0.004) (*n* = 606) (Tables 1 and 2), and such association is similarly observed in the unselected cases (Table 1). Interestingly, in the triple-negative subtype (*n* = 163), more postmenopausal patients had high PIP5K1α expression than premenopausal patients (*p* = 0.013). Overexpression of PIP5K1α was frequently associated with tumors at larger size (*p* = 0.017) and PIK3CA mutations (*p* = 0.037) (Table 3). Further, patients with higher PIP5K1α expression (*n* = 91) suffered poorer disease-free survival (DFS) as compared to the lower expression group (*n* = 72) (*p* = 0.011) (Fig. 1b). Patients with higher level of PIP5K1α (*n* = 85) also suffered poorer metastatic-free survival as compared with those with lower levels (*n* = 68), and had an increased risk of distant metastasis (HR = 2.138, 95% CI: 1.14-4.009, *p* = 0.015) (Fig. 1c). We then examined *PIP5K1A* mRNA expression in luminal and triple-negative breast cancer subtypes by using patient cohorts from database KM plotter [23]. Patients with higher *PIP5K1A* expression (*n* = 114) suffered poorer DFS than patients with lower expression (*n* = 115) (*p* = 0.048) in luminal subtype A [23] (Fig. 1d). *PIP5K1A* mRNA expression was also associated with poor DFS (*p* = 0.028) in triple-negative subtype from a patient cohort in database KM plotter [23] (Fig. 1e). There was also a clear trend that the triple-negative cancer patients from the same cohort with higher *AKT1* expression (*n* = 74) also had poorer DFS as compared to those with lower levels (*n* = 38) (*p* = 0.055), although only border significance was achieved (Fig. 1e) [23]. Gene amplifications in *PIP5K1A* were observed in 20% (*n* = 164) of unselected cases (*n* = 817 cases) (TCGA database from cBioPortal [24–26]), 17% (*n* = 102) of luminal ER^+^ subtype (*n* = 594), 24% (*n* = 29) of HER-2 positive subtype (*n* = 120) and 29% (*n* = 24) of triple-negative subtype (*n* = 82) (Fig. 1f). Thus, overexpression of PIP5K1α in breast cancer is associated with its gene amplification and poor prognosis in triple-negative breast cancer patients.

**Table 1:** Associations of PIP5K1α Expression and clinical-pathological parameters in the whole series of breast cancer (BC)

Parameters	PIP5K1α expression		Significance
	Negative/low no. (%)	Positive/high no. (%)	*P* value
*Patients’ age (years)*			
≤50	173 (49.4)	177 (50.6)	0.409
>50	286 (50.4)	281 (49.6)	
*Menopausal status*			
Premenopausal	184 (50.0)	184 (50.0)	0.516
Postmenopausal	275 (50.1)	274 (49.9)	
*Tumor stage*			
Low-grade	294 (52.0)	271 (48.0)	**0.024**
High-grade	29 (35.8)	52 (64.2)	
*NPI*			
Good (<3.4)	143 (58.6)	101 (41.4)	**0.002**
Poor (≥5.41)	64 (41.0)	92 (59.0)	

*NPI* Nottingham Prognostic Index

**Table 2:** Associations of PIP5K1α expression and clinical-pathological parameters in luminal breast cancer

Parameters	PIP5K1α expression		Significance
	Negative/low no. (%)	Positive/high no. (%)	*P* value
*Patients’ age (years)*			
≤50	102 (51.8)	95 (48.2)	0.795
>50	207 (50.6)	202 (49.4)	
*Menopausal status*			
Premenopausal	114 (53.0)	101 (47.0)	0.255
Postmenopausal	195 (49.9)	196 (50.1)	
*Grade*			
Low-grade	66 (53.2)	58 (46.8)	**0.031**
High-grade	90 (43.5)	117 (56.5)	
*Tumor stage*			
Low-stage	200 (53.1)	177 (46.9)	**0.01**
High-stage	12 (28.6)	30 (71.4)	
*NPI*			
Good ( < 3.4)	128 (57.9)	93 (42.1)	**0.004**
Poor ( ≥ 5.41)	26 (36.1)	46 (63.9)	

*NPI* Nottingham Prognostic Index

**Table 3:** Statistical association of expression of PIP5K1α and clinical-pathological parameters and the expression of PIK3CA in triple negative BC

Parameters	PIP5K1α expression		Significance
	Negative/low no. (%)	Positive/high no. (%)	*P* value
*Patients’ age (years)*			
≤ 50	42 (48.8)	44 (51.2)	0.71
> 50	36 (46.8)	41 (53.2)	
*Menopausal Status*			
Premenopausal	42 (48.3)	45 (51.7)	**0.013**
Postmenopausal	36 (47.4)	40 (52.6)	
*Tumor size*			
≤2 cm	43 (58.1)	31 (41.9)	**0.017**
>2 cm	35 (39.3)	54 (60.7)	
*PIK3CA*			
Negative	14 (70.0)	6 (30.0)	**0.037**
Positive	49 (45.4)	59 (54.6)	

### PIP5K1α inhibitor ISA-2011B suppresses tumor growth in a triple-negative breast cancer xenograft mouse model

**Fig. 2:**
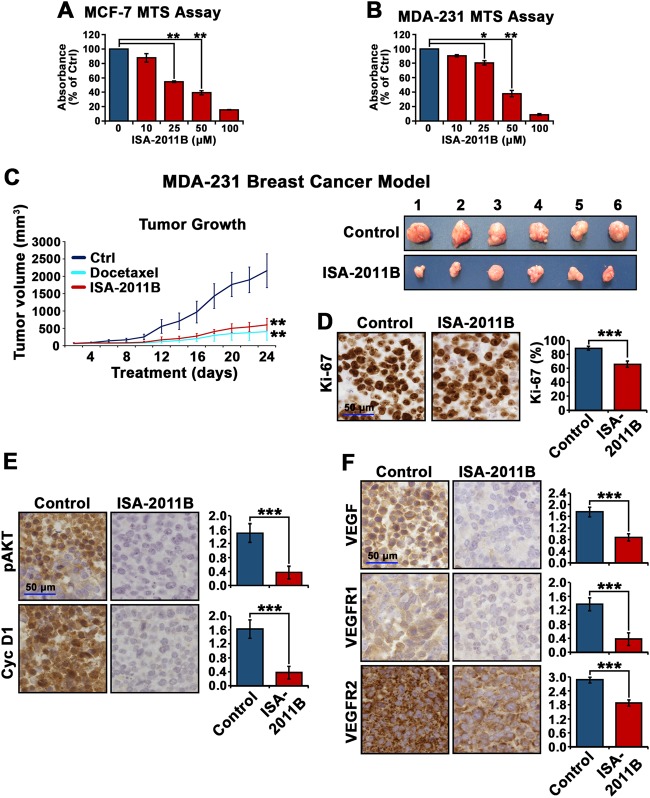
The inhibitory effect of ISA-2011B on proliferation of breast cancer cells and on growth of MDA-MB-231 xenograft tumors in mice. **a**, **b** Dose-dependent inhibitions of ISA-2011B on the proliferation of MCF-7 (**a**) and MDA-MB-231 cells (**b**) are shown. The proliferation rates were determined using tetrazolium dye-based proliferation (MTS) assay, and were shown in Y-axis by using the absorbance relative to the controls. ISA-2011B at 25 µM, 50 µM and 100 µM was used. Mean absorbance of MCF-7 after treatment with vehicle control and ISA-2011B at 25 μM respectively were 0.56 and 0.30 (difference = 45%; 95% CI = 0.02; *p* < 0.001) (**a**). Mean absorbance of MDA-MB-231 cells after treatment with vehicle control and ISA-2011B at 25 μM were 0.11 and 0.09 respectively (difference = 19.5%; 95% CI = 0.0078; *p* = 0.036). Further, the effect of ISA-2011B was stronger on MCF-7 cells at 25 µM (*p* < 0.001) than on MDA-MB-231 (*p* = 0.022). Data is presented as average of three independent experiments ( ± SD). *p* *<* 0.05, as indicated by “*”, *p* < 0.01, as indicated by “**”. **c** Growth of tumor xenografts treated with vehicle (Ctrl), docetaxel (10 mg/kg), or ISA-2011B (40 mg/kg) every second day. Treatment started on day 0 and ended on day 24 (*n* = 6 mice per group). Mean tumor volumes and upper 95% confidence intervals are shown. **P < 0.01. Tumors from each group were collected and weighed at the end of experiment. Each tumor from vehicle control-treated group and ISA-2011B-treated group is shown (right panel). **d**–**f** Representative microphotographs of immunohistochemical analysis show the expression and cellular localization of Ki-67, phosphorylated AKT, cyclin D1 (Cyc D1), VEGF, VEGFR1 and VEGFR2 in vehicle-treated tumors *vs*. ISA-2011B-treated tumors as shown in the left panels. Quantifications of proportion of Ki-67-positive and staining intensities of various biomarkers are shown in the right panels

To study the involvement of PIP5K1α in breast cancer, we tested the effect of PIP5K1α inhibitor ISA-2011B on breast cancer cell growth. Here, we treated MCF-7 and MDA-MB-231 cells with ISA-2011B at 10, 25, 50 and 100 μM doses and found a dose-dependent inhibition (ISA-2011B at 25 μM as compared with vehicle control; for MCF-7 cells, difference = 45%, 95% CI = 0.02, *p* < 0.001; for MDA-MB-231 cells, difference = 19.5%, 95% CI = 0.0078, *p* = 0.036) (Fig. 2b). Further, the effect of ISA-2011B at 25 µM was stronger on MCF-7 cells (*p* < 0.001) than on MDA-MB-231 cells (*p* = 0.022) (Fig. 2a, b).

MDA-MB-231 cell line is a triple-negative breast cancer model due to their lack of the expression of ER, HER-2 and PR, but with constitutively high expression of pAKT. To study the involvement of PIP5K1α in triple-negative breast cancer and the treatment outcome of ISA-2011B in vivo, we treated mice bearing MDA-MB-231 xenograft tumors of 50 mm^3^ in mean size with ISA-2011B, docetaxel or the vehicle control via intraperitoneal injection. After 24 days of treatment, we found ISA-2011B-treated tumors were 3-fold smaller in size as compared with the vehicle control group (mean tumor volume for vehicle-treated controls = 2160 mm^3^, mean tumor volume for ISA-2011B-treated = 600 mm^3^, difference = 1560 mm^3^; 95% CI = 0.15; *p* < 0.001, *n* = 6 per group) (Fig. 2c). Docetaxel as a positive control also showed a significant inhibitory effect on growth of MDA-MB-231 tumors (*p* < 0.001) (Fig. 2c).

Analysis of xenograft tumors removed from the mice revealed that ISA-2011B-treated tumors had significantly decreased proliferation rate as compared to the vehicle-treated controls as determined using Ki-67 expression (*p* *<* 0.001, Fig. 2d). Remarkably, expression of pSer-473 AKT was significantly decreased in ISA-2011B-treated tumors compared with controls (*p* < 0.001). ISA-2011B-treated tumors barely expressed cyclin D1, the downstream effector of AKT, and had significantly lower expression of VEGF, VEGFR1 and VEGFR2, the angiogenic factors associated with PI3K/AKT and invasiveness, as compared with that of controls (for cyclin D1, *p* < 0.001; VEGF, *p* < 0.001; for VEGFR1, *p* < 0.001 and for VEGFR2, *p* < 0.001) (Fig. 2e, f). Taken together, PIP5K1α inhibitor ISA-2011B suppressed the growth and invasiveness of triple-negative breast tumors, which is likely due to its inhibitory effect on PIP5K1α and the downstream signals in the elevated PI3K/AKT pathway.

### PIP5K1α is a key regulator of AKT pathway in MDA-MB-231 cells of triple-negative breast cancer model

**Fig. 3:**
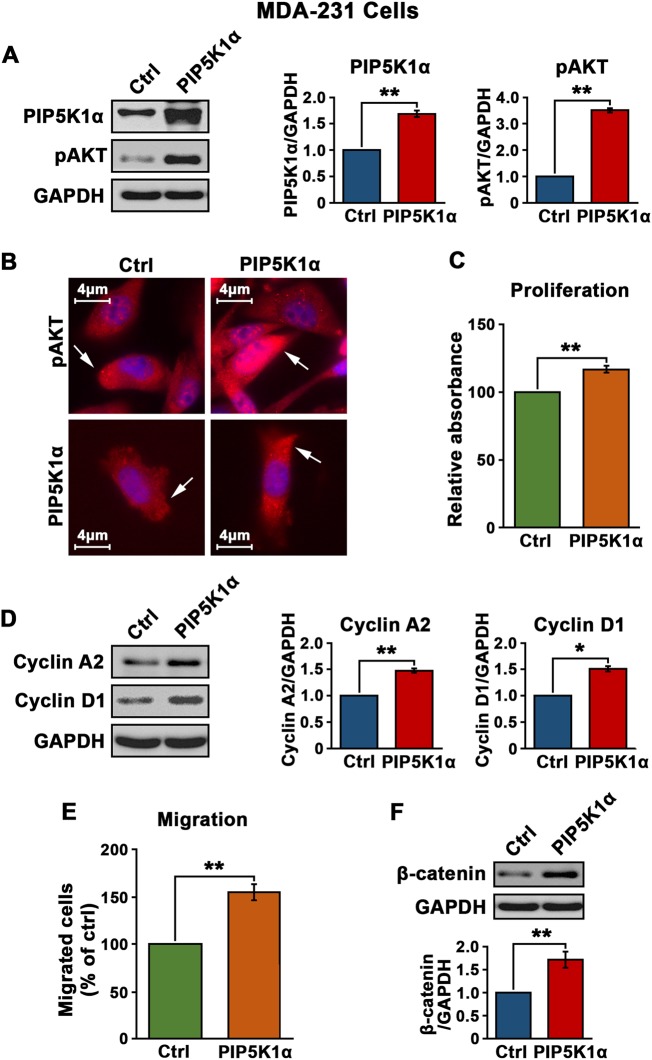
The effect of PIP5K1α overexpression on proliferation and migration in triple-negative breast cancer MDA-MB-231 cells. **a** Effect of PIP5K1α overexpression on the expression of phosphorylated AKT in MDA-MB-231 cells. Immunoblots show the expression of PIP5K1α and phosphorylated AKT in MDA-MB-231 cells transfected with pLPS-EGFP control vector (Ctrl) or pLPS-EGFP-PIP5K1α (PIP5K1α). Quantification of the immunoblots is shown. **b** Representative immune-fluorescent images show the increased expression of phosphorylated AKT (red) in MDA-MB-231 cells transfected with pLPS-EGFP-PIP5K1α (PIP5K1α) as compared with the controls. **c** The effect of PIP5K1α overexpression on the proliferation of MDA-MB-231 cells was determined using MTS assay. **d**. Immunoblots show the expression of cyclin A2 and cyclin D1 in MDA-MB-231 cells overexpressing PIP5K1α or control vector. **e** Migration assay of MDA-MB-231 cells overexpressing PIP5K1α or control vector. **f** Immunoblots show the expression of β-catenin in MDA-MB-231 cells overexpressing PIP5K1α or control vector. SD ± values indicate means of three independent experiments. *p < 0.05 and **p < 0.01 are indicated

To further elucidate the functional association of PIP5K1α with pAKT and its downstream signaling pathway, we overexpressed PIP5K1α in MDA-MB-231 cells by transfection with pLPS-EGFP-PIP5K1α or pLPS-EGFP vector as a control. PIP5K1α overexpression significantly increased the level of pSer-473 AKT by 3.5 fold in MDA-MB-231 cells as compared to those treated with the control vector (*p* < 0.001) (Fig. 3a). Immunofluorescence analysis showed the subcellular localization of PIP5K1α and pSer-473 AKT in MDA-MB-231 cells (Fig. 3b). As a result of elevated level of PIP5K1α, there was a significant increase in proliferation rate, accompanied with the increased level of cyclin D1 and cyclin A2 as compared with the controls (for proliferation, *p* < 0.001; for cyclin A2, *p* = 0.007; for cyclin D1, *p* = 0.011) (Fig. 3c, d). The migratory ability of MDA-MB-231 cells overexpressing PIP5K1α was over 50% higher than that of the control (*p* < 0.001), which was coincident with a dramatic induction of β-catenin, a key marker of invasiveness (*p* = 0.004) (Fig. 3e, f). These results suggest that PIP5K1α-induced activation of AKT may contribute to increased growth and invasiveness of triple-negative breast cancer.

### The underlying mechanisms by which ISA-2011B inhibits growth and invasiveness of MDA-MB-231 cells

**Fig. 4:**
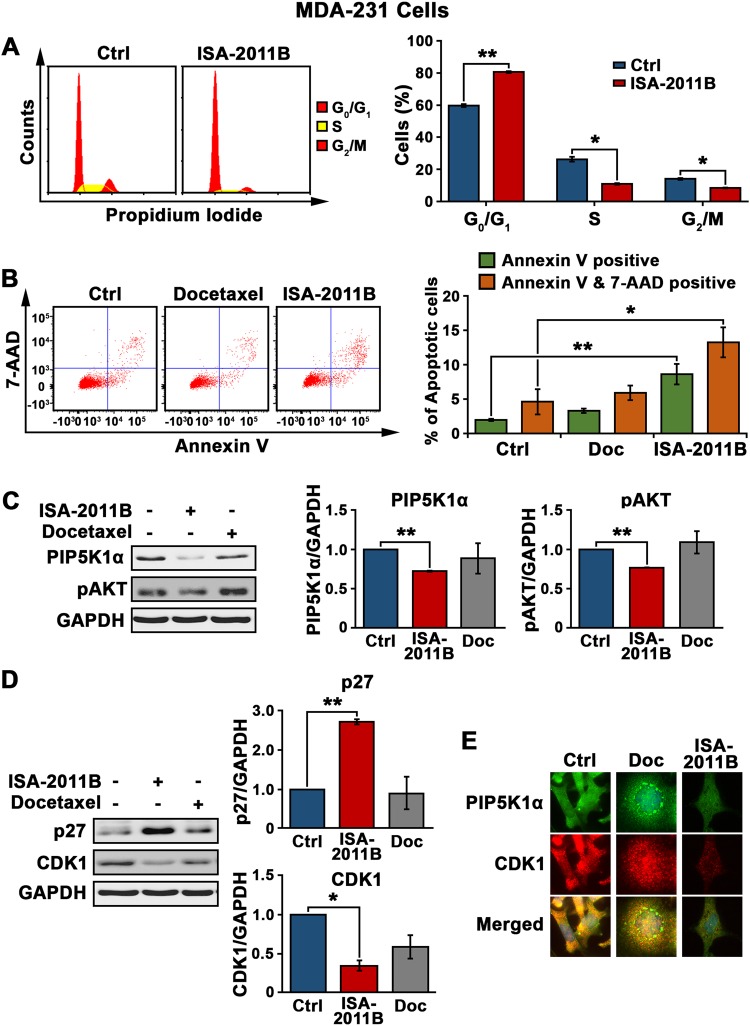
Characterization of the effect of ISA-2011B on MDA-MB-231 cells and the underlying mechanisms. **a** Cell cycle distribution of MDA-MB-231 cells treated with DMSO as vehicle control or ISA-2011B at 25 µM. Representative FACS plots show the cell cycle status in control MDA-MB-231 (Ctrl) or cells treated with ISA-2011B (ISA-2011B) (left panel). ISA-2011B-treatment reduced the proportion of MDA-MB-231 cells at S phase (mean control value = 26.31 and mean ISA-2011B-treated value = 10.94%, *p* = 0.011) and G2/M phases (mean control value = 14.025 and mean ISA-2011B-treated value = 8.47%, *p* = 0.014, as compared with the vehicle control). Data is representative of three independent experiments are indicated (right panel). **b** Representative FACS plots show the apoptosis status of MDA-MB-231 cells treated with DMSO as vehicle control, docetaxel or ISA-2011B using Annexin V-7AAD-based flow cytometry analysis (left panel). Mean percentage of early apoptosis for control 2%, mean percentage of early apoptosis for ISA-2011B was 8.6%; Difference = 7%; 95% CI = 0.53, *p* = 0.004, and the ability of ISA-2011B to induce apoptosis in MDA-MB-231 cells was greater than that of docetaxel. Data are representative of three independent experiments are indicated (right panel). **c**, **d**. Immunoblot analysis shows the effect of ISA-2011B on the expression of PIP5K1α, pAKT in the left panel (**c**) and CDK1 and p27 in (**d**). SD ± values indicate means of three independent experiments are indicated (right panel). **e** Representative microphotographs of the immuno-staining of the MDA-MB-231 cells that were treated with vehicle control, docetaxel and ISA-2011B with antibodies against PIP5K1α (green) and CDK1 (red), the merged images are shown. SD ± values indicate means of three independent experiments. *p < 0.05 and **p < 0.01 are indicated

Next, we examined functional action and mechanisms of ISA-2011B on MDA-MB-231 cells expressing elevated level of PIP5K1α. ISA-2011B-treatment reduced the ratio of MDA-MB-231 cells at S phase and G2/M phases, respectively, as compared with the ratios in the control cells (for S-phase, *p* = 0.011; for G2/M phases, *p* = 0.014) (Fig. 4a). Furthermore, a higher proportion of MDA-MB-231 cells treated with ISA-2011B underwent apoptosis as compared with the control cells or cells treated with docetaxel (the mean percentage of early apoptosis was 2% for the control, 8.6% for ISA-1011B; Difference = 7%; 95% CI = 0.53, *p* = 0.004) (Fig. 4b). ISA-2011B-treated cells had significantly lower level of PIP5K1α (*p* < 0.001) and pSer-473 AKT respectively (the average expression level of pSer-473 AKT in the control and ISA-2011B-treated cells was 0.78 and 0.60, respectively; difference = 23%, 95% CI = 0.05, *p* < 0.001) (Fig. 4c). Unlike ISA-2011B, docetaxel treatment made no difference on the expression of pSer-473 AKT (Fig. 4c). In addition, increased expression of p27 and a coincident decreased expression of CDK1, two key factors controlling cell cycle, were detected in cells treated with ISA-2011B as compared with the control cells (for p27, *p* = 0.001; for CDK1, *p* = 0.01) (Fig. 4d, e). This suggests that ISA-2011B exerts its effect on DNA synthesis and survival of cancer cells by blocking the specific pathway in breast cancer.

### Functional association of PIP5K1α, pAKT and cell cycle regulators in MDA-MB-231 cells

**Fig. 5:**
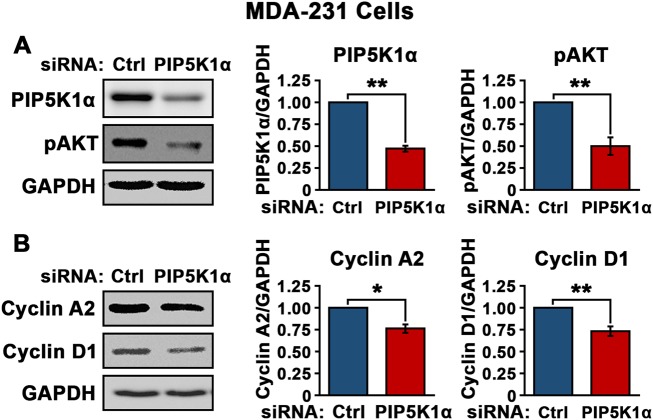
The effect of PIP5K1α inhibition on AKT pathways in MDA-MB-231 cells. *PIP5K1A* was silenced by transfecting MDA-MB-231 cells with *PIP5K1A* siRNA or scramble control (Ctrl). **a**, **b** Immunoblots for PIP5K1α, phosphorylated AKT, cyclin A2 and cyclin D1 in MDA-MB-231 cells that were transfected with *PIP5K1A* siRNA or scramble control are shown (left panel). (Mean pSer-473 AKT in control was 0.45 and 0.23 in PIP5K1α knockdown cells, difference = 0.22; 95% CI = 0.11, *p* = 0.008) SD ± values indicate means of three independent experiments. *p < 0.05 and **p < 0.01 are indicated (right panel)

Interestingly, similar to what was achieved using ISA-2011B, siRNA-mediated knockdown of *PIP5K1A* reduced PIP5K1α expression and pSer-473 AKT by over 50% as compared with the si-scramble control (*p* = 0.008) (Fig. 5a). Significantly lower level of cyclin A2 and cyclin D1 was detected in MDA-MB-231 cells expressing si-PIP5K1α as compared with the control cells (For cyclin A2, *p* = 0.038; for cyclin D1, *p* = 0.003) (Fig. 5b). Thus, inhibition of PIP5K1α via siRNA on the AKT pathway was equivalent to ISA-2011B treatment in triple-negative breast cancer. This confirmed the on-target effect of ISA-2011B in MDA-MB-231 cells.

### Regulation of PIP5K1α and its association with the estrogen-mediated pathway in luminal breast cancer

**Fig. 6:**
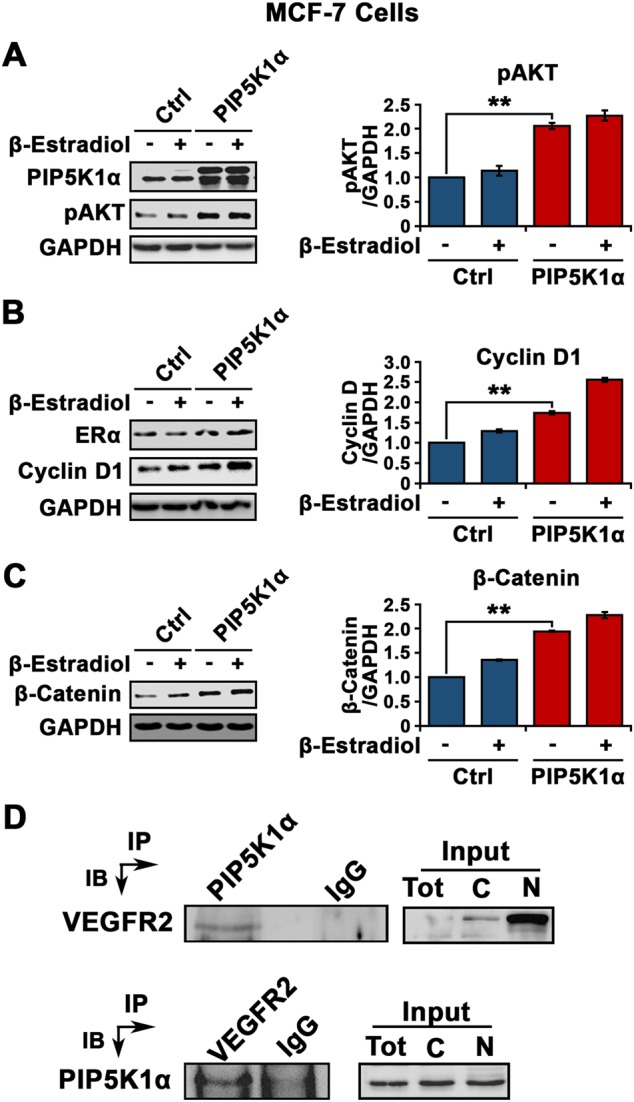
Evaluation of the effect of PIP5K1α overexpression and its functional link with estrogen signaling in MCF-7 cells. **a** Immunoblot analysis shows the effect of PIP5K1α overexpression on pAKT expression in MFC-7 cells in the presence or absence of 17β-Estradiol. Antibodies against PIP5K1α and pSer-473 AKT were used. **b**, **c** Immunoblots of cyclin D1 and β-catenin respectively in MCF-7 cells transfected with PIP5K1α or control vector in the presence or absence of 17β-Estradiol (left panels) (for cyclin D1, control transfected mean = 0.62; PIP5K1α transfected mean = 1.07, difference = 0.45; 95% CI = 0.08, *p* = 0.003; For β-catenin, control transfected mean = 0.51; PIP5K1α transfected mean = 1.0, difference = 0.49; 95% CI = 0.03, *p* = 0.0002). SD ± values indicate means of three independent experiments. *p *<* 0.05 and **p < 0.01 are indicated (right panel). **d** MFC-7 cells were subjected to immunoprecipitation (IP) assay. Antibody against PIP5K1α was used to pull down the immunocomplexes of VEGFR2 in MCF-7 cells, and antibody to IgG was used as a negative control. Conversely, antibody against VEGFR2 was also used to pull down the immunocomplexes of PIP5K1α in MCF-7 cells, and antibody to IgG was used as a negative control. Antibodies against VEGFR2 or PIP5K1α were used for immunoblot analysis (IB). The equal amount of total lysates, cytoplasmic (C) and nuclear (N) fractions were used as input control for immunoblot analysis of the immunoprecipitated protein lysates. Antibodies against PIP5K1α or VEGFR2 were used

Constitutive activation of the PI3K/AKT pathway has been found to be involved in cancer progression and drug resistance to endocrine therapy in ER^+^ breast cancer [18]. We wanted to study if PIP5K1α could regulate the PI3K/AKT pathway and whether PIP5K1α is associated with ER signaling in ER^+^ breast cancer. To this end, we overexpressed PIP5K1α in MCF-7 cells followed by the treatment of 17β-Estradiol to stimulate ERα activation. PIP5K1α overexpression increased pSer-473 AKT level by 100% as compared to the control in the presence or absence of 17β-Estradiol (average level in the control and pLPS-PIP5K1α-treated cells were 0.50 and 1.02, respectively, difference = 0.52, 95% CI = 0.13; *p* = 0.01) (Fig. 6a). The additive effects of PIP5K1α and 17β-Estradiol stimulation on upregulation of phosphorylated AKT as compared with controls, was also observed in MCF-7 cells (*p* = 0.016) (Fig. 6a). Overexpression of PIP5K1α and co-stimulation of 17β-Estradiol significantly increased expression level of cyclin D1 and β-catenin by up to 90% as compared with the control (for cyclin D1, *p* = 0.003; for β-catenin, *p* = 0.0002) (Fig. 6b and c). VEGF receptors have been shown to act as co-factors of estrogen receptor in ER^+^ MCF-7 cells [27, 28], suggesting that the VEGF signaling axis cooperates with ER signaling. We observed that PIP5K1α formed a protein complex with VEGFR2 as determined by an immunoprecipitation assay and PIP5K1α was present in both nucleus and cytoplasmic compartments in MCF-7 cells (Fig. 6d). These results suggest that PIP5K1α acts mainly on the PI3K/AKT pathway in cytoplasm and membrane, while forms a complex with VEGFR2 in the nucleus to cooperate with ERα on its target genes.

### Inhibition of PIP5K1α by ISA-2011B sufficiently blocked cyclin D1 activation and induced apoptosis

**Fig. 7:**
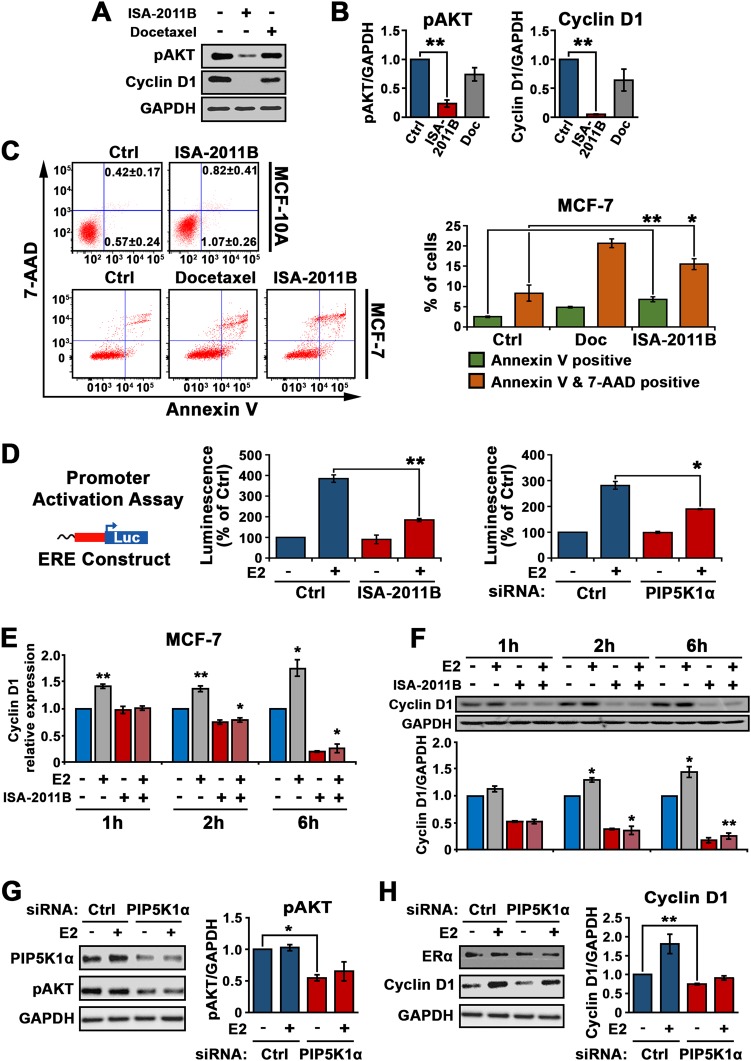
Evaluation of the effect of ISA-2011B and inhibition of PIP5K1α by siRNA-mediated knockdown on MCF-7 cells. **a**, **b** Immunoblot analysis of expression of phosphorylated AKT and cyclin D1 in MCF-7 cells treated with DMSO (-), docetaxel and ISA-2011B respectively (left panel). SD ± values indicate means of three independent experiments are indicated (right panel). **c** The effect of ISA-2011B on induction of cell death in MCF-10A cells. The percentages of Annexin V+ 7AAD- (early apoptosis) and Annexin V+7AAD+(late apoptosis) is indicated. DMSO is used as vehicle control. The effect of ISA-2011B and docetaxel on induction of cell death in MCF-7 was determined using Annexin V-7AAD-based flow cytometry analysis. Representative FACS plots show the early (green) or late apoptosis status (orange) of MCF-7 cells that were treated with DMSO as vehicle control, docetaxel or ISA-2011B (left panel). SD ± values indicate means of three independent experiments are indicated (right panel). **d** Effect of ISA-2011B treatment or si-RNA mediated knockdown of PIP5K1α on the activity of estrogen responsive gene in the presence of absence of 17β-Estradiol (E_2_) was assessed using luciferase assay. **e** Quantification of RT-PCR of *cyclin D1*, normalized with *GAPDH*, in MCF-7 cells after treatment with DMSO (-), E_2_, ISA-2011B and combination of E_2_ and ISA-2011B for 1 h, 2 h and 6 h. **f** Western blot analysis of Cyclin D1 and GAPDH in MCF-7 cells after treatment with DMSO (-), 17β-Estradiol, ISA-2011B and combination of E_2_ and ISA-2011B for 1 h, 2 h and 6 h. Below panel indicates the semi-quantification of data. **g**, **h** The effect of siRNA-mediated knockdown of PIP5K1α on the expression of pAKT and cyclin D1 in presence or absence of E_2_. SD ± values indicate means of three independent experiments. *p < 0.05 and **p < 0.01 are indicated

To test our hypothesis as mentioned above, we first investigated the effect of ISA-2011B treatment on PI3K/AKT and ER signaling pathways. ISA-2011B treatment in MCF-7 cells reduced pSer-473 AKT level by approximately 40% and cyclin D1 level by over 90% as compared with the control cells (for pSer-473 AKT, *p* = 0.007; for cyclin D1, *p* < 0.0001) (Fig. 7a, b). In contrast, docetaxel treatment did not alter the expression level of phosphorylated AKT and cyclin D1 (Fig. 7a, b). In addition, ISA-2011B treatment had no pronounced effect on apoptosis in non-malignant MCF-10A breast cells as assessed by flow cytometry and immunoblot analysis of activated PAPR, a pro-apoptotic marker (Fig. 7c and Fig. 1 in Supplemental Figures and Legends). ISA-2011B induced a high degree of apoptosis in MCF-7 cells, which was similar to that induced by docetaxel treatment, as measured by flow cytometry analysis (early apoptosis control mean value = 2.5, ISA-2011B mean value = 6.8; docetaxel mean value = 4.9; ISA-2011B *vs* control *p* *=* 0.0005; for ISA-2011B *vs* Docetaxel *p* = 0.02) (Fig. 7c). Immunofluorescent analysis of apoptotic nuclei of the cells and immunoblot analysis of activation of PARP further revealed that ISA-2011B induced apoptosis, rather than necrosis in MCF-7 cells (Supplemental Fig. 2).

Next, to investigate whether PIP5K1α may cooperate with ERα to regulate its target genes, we performed assays to test the outcome of ISA-2011B inhibition or *PIP5K1A* knockdown on ERα-mediated estrogen signaling, using luciferase (Luc) reporter under the control of an estrogen responsive element (ERE) [29]. Treatment of MCF-7 cells harboring a luciferase reporter containing 3 consensus EREs, with 17β-Estradiol followed by the treatment with ISA-2011B or DMSO vehicle control was performed. As expected, 17β-Estradiol treatment induced ERE reporter luciferase activity by 300% in MCF-7 cells as determined by luciferase activity assays (*p* = 0.004) (Fig. 7d). In contrast, ISA-2011B treatment abrogated 17β-Estradiol-induced transcriptional activity of the genes targeted by ERα with 200% reduction (*p* = 0.009) (Fig. 7d). Similar to ISA-2011B, *PIP5K1A* knockdown exerted equivalent inhibition on 17β-Estradiol-triggered transcriptional activity of ERα target genes (*p* = 0.027) (Fig. 7d). This suggests that the induction of ERα target genes by 17β-Estradiol can be suppressed by both ISA-2011B and the knockdown of *PIP5K1A*. Next, we treated MCF-7 cells with 17β-Estradiol and ISA-2011B individually or in combination for 1, 2 and 6 h, as the cell cycle of MCF-7 cells has been relatively unperturbed prior to the longer treatment. We observed that the 17β-Estradiol-induced upregulation of both mRNA and protein expression of cyclin D1, a direct target of ERα, was abrogated by ISA-2011B treatment readily at 2 h and up to 6 h (for cyclin D1 mRNA, *p* = 0.017 at 2 h and *p* = 0.01 at 6 h; for cyclin D1 protein, *p* = 0.004 at 2 h and *p* = 0.002 at 6 h) (Fig. 7e, f). In addition, knockdown of*PIP5K1A* resulted in a significant reduction of pSer-473 AKT by 50% as compared to the control (Fig. 7g, difference = 0.31; 95% CI = 0.06, *p* = 0.011). The level of cyclin D1 was significantly lower in MCF-7 cells expressing siPIP5K1α than in the control cells (*p* = 0.006). These results showed that the inhibitory effect on activation of AKT and expression of cyclin D1 by PIP5K1α siRNA knockdown was equivalent to ISA-2011B treatment. Further, depletion of PIP5K1α also blocked 17β-Estradiol-induced cyclin D1 expression (Fig. 7h). The ability of ISA-2011B to inhibit 17β-Estradiol-ERα downstream gene activity such as cyclin D1 was found to be similar to that of si-PIP5K1α. Taken together, PIP5K1α inhibition, either by ISA-2011B or siRNA-mediated knockdown, significantly abrogated the activity of ERα and estrogen on inducing the target genes, suggesting a potential of ISA-2011B for targeting ER^+^ breast cancer with elevated PIP5K1α expression.

## Discussion

Constitutively activated PI3K/AKT pathway resulted from frequent *PIK3CA* gene mutations has been linked to different types of human breast cancers [18]. Previous studies have shown PIP5K1α as an emerging cancer drug target and a biomarker in prostate cancer, and a small molecule PIP5K1α inhibitor with the ability to suppress tumor growth in a castration-resistant prostate cancer xenograft mouse model [15, 16]. The mechanistic studies have shown that PIP5K1α acts upstream of the PI3K/AKT pathway as a lipid kinase to produce PIP2, an important molecule to activate AKT by PI3K in this signaling pathway [12, 30].

In this study, we show that PIP5K1α may be able to play a significant role in breast cancer progression and metastasis. Overexpression of PIP5K1α was associated with low DFS and increased risk of distant metastasis in triple-negative breast cancer. In addition, high level of PIP5K1α protein was linked to luminal breast cancer subtype with high-grade and poor prognosis. Furthermore, elevated level of *PIP5K1A* mRNA was associated with poor DFS in luminal A subtype of breast cancer. Our study was the first to show the clinical significance of PIP5K1α in breast cancer subtypes, particularly in the triple-negative breast cancer.

Our findings unravel important roles PIP5K1α may play in proliferation, survival and metastasis of the triple-negative breast cancer by using MDA-MB-231 cell line and in vivo xenograft mouse model. Our results showed that PIP5K1α overexpression significantly promoted proliferation and migratory ability of MDA-MB-231 cells, and such effect in breast cancer was similar to what was found in prostate cancer cell lines such as LNCaP and PC-3. We further demonstrated that PIP5K1α exerts its effect on the PI3K/AKT pathway, which in turn activates the downstream effectors such as cyclin A2, cyclin D1 and β-catenin. As in prostate cancer, PIP5K1α plays such a role in breast cancer via its kinase activity to produce PIP2, which activates the PI3K/AKT pathway.

Patients with triple-negative breast cancer often experience worst clinical outcome, and currently no effective targeted therapies are available for treatment. In our current study, we demonstrated that PIP5K1α inhibitor, ISA-2011B, could induce apoptosis, with an effect comparable to docetaxel. In addition, it significantly suppressed growth of highly invasive MDA-MB-231 tumor in xenograft mice, which serves as a clinically relevant triple-negative breast cancer model. Unlike docetaxel, which is a cytotoxic drug targeting all proliferative cells, ISA-2011B inhibits tumor growth and promotes apoptosis by blocking PI3K/AKT, a key cancer survival and invasion pathway in MDA-MB-231 cells. In our studies, PIP5K1α overexpression remarkably increased the level of phosphorylated AKT, while ISA-2011B treatment or PIP5K1α knockdown significantly decreased phosphorylated AKT, leading to down-regulation of the downstream effectors. We further confirmed the effect of ISA-2011B in vivo in MDA-MB-231 xenograft tumors. ISA-2011B not only strongly inhibited tumor growth, but also significantly lowered expression of phosphorylated AKT and its downstream effectors such as cyclin D1, VEGF, VEGFR1 and VEGFR2. In striking contrast, docetaxel treatment did not affect expression of AKT and its effectors in the pathway. Thus, our study revealed the potential of PIP5K1α as a druggable-target. In addition, it shows the therapeutic potential of PIP5K1α inhibitor ISA-2011B, as it specifically induces apoptosis and inhibits metastasis by blocking the elevated PI3K/AKT pathway in triple-negative breast cancer.

It has been reported that abnormal activation of PI3K/AKT frequently occurs in a high proportion of ER^+^ breast cancer patients, who often suffer from cancer recurrence and bone metastasis [3]. In this study, we demonstrated that abnormally high expression of the PIP5K1α/AKT pathway led to increased survival and invasiveness of MCF-7 cancer cells. The effect of ISA-2011B on MCF-7 cancer cells was mediated through the PI3K/AKT pathway. Since the oncogenic mutations of PIK3CA are found to trigger luminal ER^+^PR^+^ tumors in a transgenic mouse model [31], constitutively activated PI3K/AKT pathway may cooperate with ERα to promote invasiveness of ER^+^ breast cancer. Our study showed that ISA-2011B blocked the activity of 17β-Estradiol to induce expression of ERα target genes, leading to lower expression level of cyclin D1 and CDK1 in MCF-7 cells. ISA-2011B abrogated an early effect of 17β-Estradiol on cyclin D1 expression in MCF-7 cells at 2 h and up to 6 h. Although the cell cycle in MCF-7 cells was relatively unperturbed prior to the longer treatment, early ERα-target upregulation by 17β-Estradiol was impaired after ISA-2011B treatment.

We found a protein–protein complex formation between VEGFR2 and PIP5K1α. ISA-2011B or PIP5K1α knockdown dramatically reduced expression of 17β-Estradiol-ERα induced target genes such as the VEGF axis [27, 28]. Further, the 17β-Estradiol-ERα complex regulates expression of target gene by binding directly to estrogen response elements (EREs) [32, 33]. Thus, in addition to its role as a lipid kinase to produce PIP2, PIP5K1α may also act as a co-factor of VEGFR2 to regulate the transcriptional activity of ERα-target genes through VEGFR2. Thus, our study uncovers several unrecognized inter-links among PIP5K1α, PI3K/AKT, ER and cyclin D1 in ER^+^ breast cancer. Furthermore, we showed that ISA-2011B did not exert toxic effect on a well-characterized normal breast cell line MCF-10A in striking contrast to MCF-7 cancer cells. Thus, ISA-2011B has an on-target inhibitory effect on breast cancer cells. Our study demonstrated the potential of developing ISA-2011B as a new targeted therapy to treat triple-negative and ER^+^ breast cancers with metastatic potential.

## Materials and methods

### Sources of tissue specimens, TMAs and mRNA expression data

TMAs containing a well-characterized annotated series of unselected primary breast cancer from 913 patients were constructed as described before [22] (Tables 1, 2 and 3). Distant metastasis-free survival or DFS were analyzed. The detailed clinico-pathological data and treatment regimens were described in previous reports [34, 35]. For evaluation of tumor tissues on TMAs, all of the samples were coded and scored by pathologists and scientists working at different institutions. Immunohistochemical analysis was performed as previously descried [16]. The second (*n* = 112) and third (*n* = 229) cohorts used came from the Kaplan–Meier plotter (KM Plotter) (www.kmplot.com) [23], and the fourth cohort (*n* = 817) was obtained from TCGA in the cBioPortal database [24–26]. The study was approved by the Nottingham Research Ethics Committee and the Ethics Committee at Lund University and Region Skåne in Sweden. The guidelines in Helsinki Declaration of Human Rights were strictly followed during the study.

### Cell culture and treatments

Breast cancer cell line MDA-MB-231 and MCF-7 were purchased from American Type Culture Collection (Manassas, VA) and cultured in RPMI-1640 medium with phenol red, which was supplemented with 10% fetal bovine serum (FBS), 1% penicillin-streptomycin-neomycin and 2 mM L-Glutamine (PAA Laboratories, GmbH). Before treatment, the cells were cultured in phenol red-free RPMI-1640 medium supplemented with 10% charcoal-stripped serum for 24 h. ISA-2011B at 20 µM or 50 µM, Docetaxel at 10 nM, solvent of 0.1% DMSO (V/V), or 17β-Estradiol at 10 nM were used for the treatment.

### PIP5K1α transfection and siRNA knockdown

Full-length cDNA of human PIP5K1α (pLPS-PIP5K1α) or control vector (pLPS-EGFP) were constructed and transiently transfected using a transfection reagent Lipofectamine^®^ 2000 (Life Technologies, Paisley, UK) following the manufacturer’s instructions [16]. In PIP5K1α knockdown experiment, breast cancer cells were treated with *PIP5K1A*-siRNA or negative control siRNA (VWR International Inc.) respectively by using TransIT-TKO^®^ kit according to the manufacturer’s instructions (Mirus Bio LCC). Various analyses were performed on the cells at the time points of 24, 48 and 72 h post-transfection.

### Establishment of MDA-MB-231 xenograft mouse model and treatment

All in vivo experiments were performed after approval by the local ethics committee at Lund University. MDA-MB-231 cells (4 × 10^6^) were implanted subcutaneously into the female BALB/c nude mice at the age of 8–12 weeks. After the mean tumor volumes reached 50 mm^3^, the mice were randomly assigned into three different groups (6 mice/group). The three groups of mice were treated with vehicle (control), docetaxel (10 mg/kg) and ISA-2011B (40 mg/kg), respectively, by intraperitoneal injection once every other day. The body weight and tumor diameters were measured every other day. The tumor volume was calculated with tumor diameters using the equation (*a* × *b*^2^/2). After the treatment was completed, the mice were sacrificed and the tumors were collected for analysis.

### Immunohistochemical analysis

Breast cancer TMA and paraffin-embedded tumor tissues from the xenograft study were stained using various antibodies as previously described [16]. The slides were stained using a semiautomatic staining instrument (Ventana ES) and then scanned with a high-resolution scanner (ScanscopeCS, Aperio). The stained TMAs were scored with semi-quantitative Histochemical score (H-score), which was determined by multiplying the percentage of stained invasive tumor cells (minimum 0 and maximum 100) and the staining intensity (0 was negative, 1 was weak, 2 was moderate and 3 was strong staining). All of the specimens were blindly scored using a coding system.

### Immunoblot, immunoprecipitation and subcellular fractionation assays

Immunoblot, immunoprecipitation and subcellular fractionation were performed as described previously [15]. For immunoprecipitation assay, PIP5K1α or VEGFR2 antibody was used to pull down the immune-complex, with an IgG antibody (BD Biosciences, San Jose, CA, USA) used as negative control. Various antibodies were used in immunoblot as shown below: PIP5K1α (Proteintech Inc. and Cell Signaling Technology), Phospho-S473 AKT, CDK1, cyclin D1 (Cell Signaling Technology and Santa Cruz Biotechnology), VEGF, VEGFR1, VEGFR2, p27, Cyclin A2, anti-GAPDH (Santa Cruz Biotechnology), ERα (Biosite), Ki-67 (DAKO), MMP-9 (Abcam), anti β-Actin (MP Biochemicals), cyclin E (Upstate Inc.). Immunoblot analysis was performed and semi-quantified using ImageJ Image Analysis Software (NIH, MD, USA).

### Immunofluorescence assasy

Breast cancer cells were first seeded on glass coverslips and then treated with different agents for 48 h. Image-iT™ FX signal enhancer (Molecular Probes, Inc) was used for blocking nonspecific background staining. Phospho-473 AKT, PIP5K1α and VEGFR2 antibodies were used as primary antibodies. Donkey anti-rabbit conjugated to Rhodamine antibody (Chemicon/Millipore) or donkey anti-goat conjugated to FITC antibody at 1:200 dilution, and goat anti-rabbit conjugated to Alexa Fluor 488 at 1:500 dilution (Invitrogen) were used as secondary antibodies. 4′,6-Diamidino-2-phenylindole (SERVA Electrophoresis GmbH) was used to counterstain the cells for visualization of cell nuclei. The images were viewed and taken using Olympus AX70 fluorescent microscope (Nikon DS-U1).

### Flow cytometry (FACS)-based apoptosis and cell cycle analysis

Flow cytometry (FACS)-based assays were performed on cells treated with ISA-2011B to analyze cell cycle and apoptosis. Propidium iodide (Sigma-Aldrich) staining was used during flow cytometry for cell cycle analysis (CyAn ADP, Beckman Coulter). For apoptosis assay, FITC or PE-conjugated Annexin V and 7-AAD staining was performed according to the manufacturer’s protocol (BD Biosciences). Data analysis was performed with FCS Express (DeNovo Software), FlowJo (Tree Star Inc., OR, USA) or CytExpert (Beckman Coulter) software.

### RNA purification and PCR

mRNA was purified from MCF-7 cells after treatment as previously described [36]. The following primers were used in PCR: CCND1 forward: 5′-ATG CCA ACC TCC TCA ACG AC-3′, reverse: 5′-TCT GTT CCT CGC AGA CCT CC-3′. GAPDH forward: 5′-AAC AGC GAC ACC CAC TCC TC-3′, reverse: 5′-GGA GGG GAG ATT CAG TGT GGT-3′. PCR was performed under the following cycling condition: DNA denaturation for 5 min at 95 °C, 18 cycles of 30 s annealing at 65.5 °C and 30 s extension at 72 °C, and final 10 min extension at 70 °C. Semi-quantification was performed using ImageJ Image Analysis Software (USA).

### Luciferase assay

MCF-7-ERE-luc cells were cultured in phenol red-free 10% charcoal stripped medium in the presence of various agents alone or in combination. 10 nM 17β-Estradiol, alone or in combination with either 50 µM ISA-2011B or 0.1% DMSO (V/V) solvent, were used for treatment. After the treatment, the cells were first lysed and then measured for Firefly Luciferase and Renilla Luciferase activities, which were tested by using the dual luciferase reporter assay kit (Promega). The effect of ISA-2011B or si-PIP5K1α on transcriptional activity of the ER target genes containing ERE in MCF-7-ERE-luc cells was measured with Infinite^®^ M200 multimode microplate reader equipped with a dual injector (Tecan Sunrise™).

### Cell migration assay

Cell migration assay was performed in Boyden trans-well chambers (8 μm) following the manufacturer’s instructions (BD Biosciences). The cells were seeded into the upper chamber, while 20% of FBS as chemo-attractant was applied in the lower chamber. After 20 h of incubation, the cells that did not migrate and remained in the upper chamber were removed with cotton swabs, and the cells migrated away were first fixed with 4% paraformaldehyde, and then stained with crystal violet dye. The stained cells were quantified under microscope.

### Statistical analysis

Tukey-test, T-test, Kruskal Wallis/ANOVA test and Spearman Rank Correlation Test were performed. The immunohistochemistry H-scores were log-transformed to compensate for the non-normal distribution of H score data. The mean value is the average value of all samples. The standard deviation (SD) is an indication of variability of all samples. The precision of the sample mean is indicated by standard error. The confidence level is expressed using 95% confidence interval (CI). All of the statistical testes were two-sided, and *p* < 0.05 was considered to be statistical significant. For all in vitro experiments, at least three independent experiments were conducted. All quantification data presented in the studies was average of at least three independent experiments. Statistical software and Social Science software (SPSS, version 21, Chicago) were used in statistical analysis. Detailed statistical calculations were described in relevant figure text.

## Electronic supplementary material

Supplemental Materials
