# Supplementary material for: The role of PIP5K1α/pAKT and targeted inhibition of growth of subtypes of breast cancer using PIP5K1α inhibitor
Source: Oncogene. 2018 Aug 13;38(3):375–89. doi: 10.1038/s41388-018-0438-2 (PMC6336681; doi:10.1038/s41388-018-0438-2)
Supplement: Supplementary file 1 — Supplemental Materials [file 41388_2018_438_MOESM1_ESM.docx]

**Supplemental Figures and legends**

**Supplementary Figure 1.** Western blot evaluation of apoptosis by the expression of cleaved (cl-PARP) in normal breast epithelial cells (MCF-10A) and breast cancer cells (MCF-7) after treatment with DMSO or ISA-2011B.


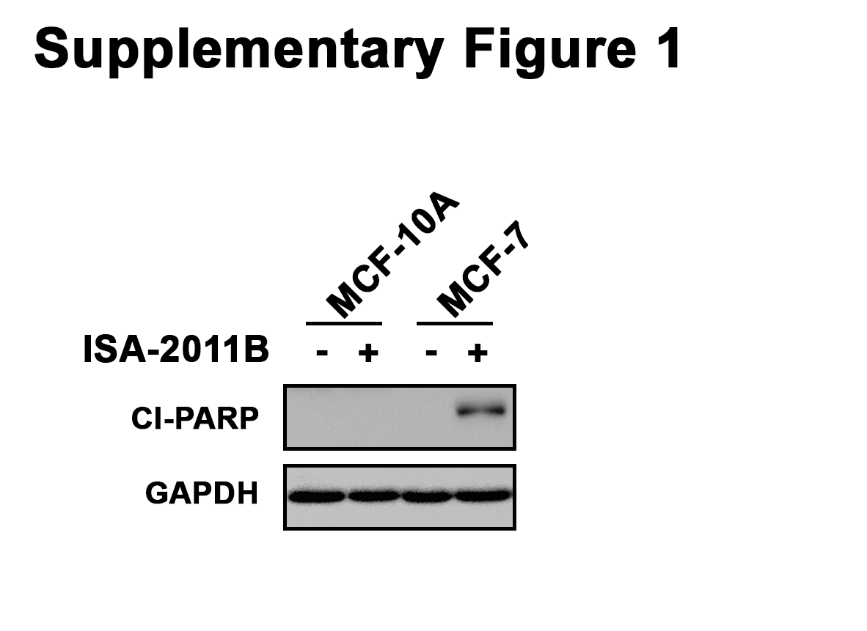


**Supplementary Figure 2: Effect of ISA-2011B and Docetaxel in MCF-7 cells. A.** MCF-7 cells that were treated with ISA-2011B or docetaxel were stained with propidium iodide (red); apoptotic nuclei are indicated by arrows. (B) Immunoblots of cl-PARP in MCF-7 cells treated with DMSO, docetaxel or ISA-2011B.


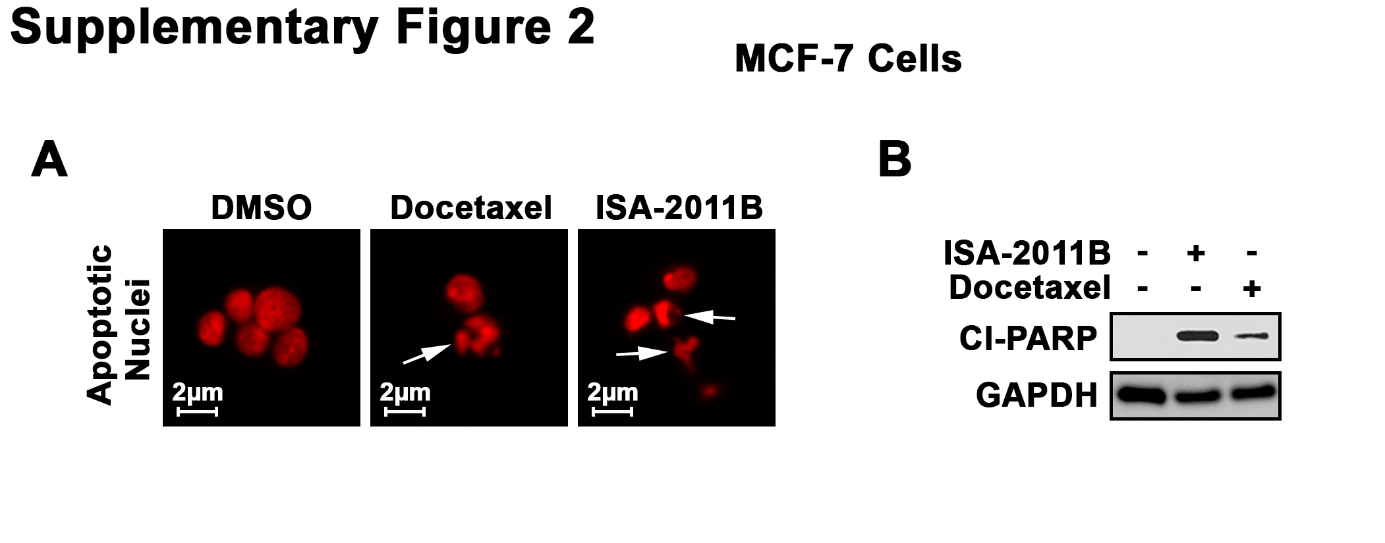


**Materials and Methods**

*Tissue Specimens, Tissue Microarrays and mRNA expression data*

Tissue microarrays (TMAs) containing a well-characterized annotated series of unselected primary breast cancer from 913 patients were constructed as previously described (1). Distant metastasis-free survival (DMFS) or disease-free survivals (DFS) were determined. The time from the surgery to the first event of distant metastasis or disease-recurrence was taken into account. The detailed clinical-pathological data and treatment regimens are indicated in (2, 3). This study was performed after approval by the Nottingham Research Ethics Committee 2 under the title “Development of a molecular genetic classification of breast cancer”. The second patient cohort (n=112) was extracted from the publically available integrative data analysis tool, termed the KM Plotter ([www.kmplot.com](http://www.kmplot.com)). The DFS of triple negative breast cancer patients was assessed based on the expression of *PIP5K1A* or *AKT1* expression (4). Somatic amplification or alterations in *PIP5K1A* gene were examined in a patient cohort (n=817) using dataset (TCGA database) in the cBioPortal database Comprehensive Molecular Portraits of Invasive Lobular Breast Cancer (cBioPortal, (5-7)). The study was also approved by the Ethics Committee at Lund University and Region Skåne in Sweden. The Helsinki Declaration of Human Rights was strictly observed.

*Immunohistochemical Analysis*

Tumor tissue microarrays were stained as previously described (8). The semiautomatic staining instrument (Ventana ES, Ventana Inc., Tucson, AZ) was used for staining procedure. Stained tissue slides were scanned using a high resolution scanner (ScanscopeCS, Aperio, Vista, CA), and photomicrographs were taken at magnifications of 20X or 40X. Stained TMAs were scored using semi-quantitative H-score (Histochemical score), determined by multiplying the percentage of the invasive tumor cells stained (minimum 0 and maximum 100) by the intensity of the staining (where 0 was negative, 1 was weak, 2 was moderate and 3 was strong staining). In this scoring approach, only the cytoplasmic staining in invasive tumour cell was considered. All cases were scored blinded to clinico-pathological and outcome data.

*Cell Culturing and Treatments*

The MDA-MB-231 cell line and MCF-7 cell line were purchased from American Type Culture Collection (Manassas, VA) and were maintained in phenol red-containing RPMI-1640 medium supplemented with 10% fetal bovine serum (FBS), 1% penicillin-streptomycin-neomycin and 2 mM L-Glutamine (PAA Laboratories, GmbH, Austria). For treatment, cells were grown in phenol red-free RPMI-1640 medium containing 10% charcoal stripped-serum for 24 hours. Cells were then treated with drugs alone or in combination for 24 hours or 48 hours. ISA-2011B at 20 µM or 50 µM final concentrations or solvent 0.1% DMSO (V/V) was used. For 17β-Estradiol (E_2_) treatment, a final concentration of 10 nM was used for treatment of MCF-7 cells.

*Plasmids, Stable Transfection, and siRNA Knockdowns*

Full-length human PIP5K1α cDNA (pLPS-PIP5K1α) or control vector (pLPS3-EGFP) were transiently transfected using Lipofectamine® 2000 transfection reagent (Life Technologies, Paisley, UK) according to the manufacturer's instructions (8). The knock-down experiments were performed using siRNAs against *PIP5K1A* or negative control (VWR International Inc.) in breast cancer cells using TransIT-TKO® kit according to manufacturers’ protocol (Mirus Bio LCC). Cells were then collected after 24, 48 and 72 hours post-transfection.

*Mouse models of xenograft* MDA-MB-231 *tumors and treatment*

All *in vivo* experiments were performed after approval from the local ethics committee at Lund University. BALB/c nude mice aged 8–12 weeks were used in the experiments. 4 × 10^6^ MDA-MB-231 cells were implanted into the BALB/c nude mice aged 8–12 weeks (6 mice/group). Tumor xenografts were treated with vehicle (control), docetaxel (10 mg/kg), ISA-2011B (40 mg/kg) by intraperitoneal injection every second day. Weights of the animals and tumor volume were recorded regularly. Tumor diameters were measured using calipers, and volumes were calculated using the equation (axb^2^/2), where ‘a’ and ‘b’ represent the larger and smaller diameters, respectively. Tumor samples were collected post-mortem and used for immuno-histochemical analyses.

*Proliferation Assay*

Cell proliferation assay was performed using MTS proliferation reagent (Promega Biotech) according to manufacturer’s protocol. Transfected cells at 5 x 10^3^/well were cultured in 96-well plates for 48 hours. Viability was determined by measuring the absorbance at 490 nm wavelength, on Infinite® M200 multimode microplate reader (Tecan Sunrise™).

*Immunoblot analysis and source of antibodies*

Immunoblot analysis was performed as described earlier (8). Cells or tumor tissues were harvested and lysed in ice-cold RIPA buffer. Protein (20-30 μg) were separated on 12% SDS-PAGE gels and transferred onto nitrocellulose membranes. Enhanced ChemiLuminescence detection system (Pierce, Rockford, USA) was used for signals visualization, and documented with an AlphaImager CCD system. Immunoblots were semi-quantified using ImageJ Image Analysis Software (NIH, Baltimore, USA) and data was represented as fold change relative to control (β-actin or GAPDH). The following antibodies were used in this study: PIP5K1α #15713-1-AP (Proteintech Inc., and Cell Signaling technology), Phospho-473 AKT #4060 and cyclin D1 (Cell Signaling technology and Santa Cruz Biotechnology), VEGF #SC-152, VEGFR1 #[SC-271789](https://www.scbt.com/scbt/sv/product/flt-1-antibody-d-2?requestFrom=search), VEGFR2, p27, Cyclin A2, anti-GAPDH (Santa Cruz Biotechnology Inc. CA), ERα #AMB-7491 (Biosite), Ki-67 #M7240 (DAKO, Glostrup, Denmark), MMP-9 #ab38898 (Abcam, Cambridge, UK), anti β-Actin (MP Biochemicals, Illkirch, France), cyclin E (Upstate Inc.), CDK1 #610038 (BD Transduction Lab Inc.) and β-catenin (Zymed, ThermoFisher Inc), HRP-conjugated anti-mouse IgG or anti-rabbit IgG secondary antibodies (GE Healthcare).

*Immunoprecipitation and subcellular fractionation*

Immunoprecipitation was prepared as previously described (8). Briefly, freshly prepared protein lysates were incubated with antibodies against PIP5K1α or VEGFR2 with 30 μl of G-sepharose beads (GE Healthcare) for 3 hours at 4 °C to pull down the immune-complexes. Antibody against IgG (BD Biosciences, San Jose, CA, USA) was used as a negative control. The immune-complexes were subsequently washed in RIPA buffer and subjected to immunoblot analysis. Subcellular fractionation was performed as previously described (9). For obtaining the nuclear fraction, the pellets were incubated in the ice-cold nuclei isolation buffer (10 mM HEPES pH 7.9, 1,5 mM MgCl_2_, 10 mM KCl, 0.5 mM DTT, 1 % Triton X-100, 15 % protease inhibitor cocktail Complete Mini, 1 mM PMSF). After the separation steps, the nuclear and cytoplasmic fractions were subjected to immunoblot analysis.

*Immunofluorescence analysis*

Breast cancer cells were grown on the glass coverslips for 24 hours and were then treated with the indicated drugs for 48 hours. Cells were fixed with 4% paraformaldehyde in PBS. Image-iT™ FX signal enhancer (Molecular Probes, Inc) was used for blocking unspecific background staining. Primary antibodies against Phospho-473 AKT, PIP5K1α and VEGFR2 were used. The secondary antibodies including donkey anti-rabbit conjugated to Rhodamine (Chemicon/Millipore International Inc, Temecula, CA, USA) or anti-goat conjugated to FITC antibodies at 1:200 and goat anti-rabbit Alexa Fluor 488 at 1:500 (Invitrogen, Stockholm, Sweden) were used. 4′,6-Diamidino-2-phenylindole counterstain (SERVA Electrophoresis GmbH, Heidelberg, Germany) was used to visualize cell nuclei. The slides were imaged under an Olympus AX70 fluorescent microscope (Nikon DS-U1, Stockholm, Sweden).

*FACS-Based Cell Cycle Analysis and Apoptosis Assay*

For cell cycle analysis, ethanol fixed cells (overnight at -20 °C) were centrifuged, washed with PBS and stained with propidium iodide (Sigma-Aldrich). DNA content was measured with flow cytometry (CyAn ADP, Beckman Coulter). For apoptosis analysis, treated cells were stained with FITC or PE-conjugated Annexin V and 7-AAD according to the manufacturers’ Protocol (BD Biosciences). Data was analyzed using FCS Express (DeNovo Software, CA, USA), FlowJo (Tree Star, Inc., OR, USA) or CytExpert (Beckman Coulter, FL, USA) softwares.

### *Luciferase Assays*

MCF-7-ERE-luc cells were freshly subcultured in RPMI-1640 supplemented with 10% FBS. Cells were treated with drugs alone or in combination for further 24 hours in phenol red-free 10% charcoal stripped medium. 10 nM 17β-Estradiol (E_2_) alone or together with ISA-2011B at 50 µM final concentrations or solvent 1% DMSO (V/V) were used. Cells were lysed and Firefly Luciferase and Renilla Luciferase activity was determined with the dual luciferase reporter assay kit (Promega) according to the manufacturers’ protocol. Luciferase activity was determined using an Infinite® M200 multimode microplate reader (Tecan Sunrise™), equipped with dual injector.

*Migration Assay*

The Boyden trans-well chambers (8 μm) were used for migration assay according to the manufacturer's protocol (BD Biosciences). Briefly, cells were seeded into the upper chamber and 20% FBS was used in the lower chamber as chemo-attractant. After 20 hours incubation, non-migrated cells were removed with cotton swabs and the migrated cells were fixed with 4% paraformaldehyde, stained with crystal violet dye and counted under a microscope.

### *RNA isolation and RT–PCR*

RNA was isolated from MCF-7 cells after treatment as previously described (10). RNA was reversely transcribed to cDNA following the supplier’s protocol (ThermoFisher Scientific). The following primers were used: CCND1 – forward: 5' - ATG CCA ACC TCC TCA ACG AC –3'; and reverse: 5' - TCT GTT CCT CGC AGA CCT CC –3'. GAPDH–forward: 5′- AAC AGC GAC ACC CAC TCC TC -3′ and reverse: 5′- GGA GGG GAG ATT CAG TGT GGT -3′. The PCR conditions were: denature the DNA at 95° C for 5 min, followed by denaturing at 98°C for 30 secs, 65.5°C for 30 secs and extension at 72°C for 30 secs with 18 cycles and final extension at 70°C for 10 min. Semi-quantifications were performed using ImageJ Image analysis software (NIH, MD, USA).

*Statistical Analysis*

Tukey-test, *T-test,* Kruskal Wallis/ANOVA test and Spearman rank correlation tests were performed. The immunohistochemistry H-scores were log-transformed to overcome the non-normal distribution of H score data. All statistical testes were two-sided, and *p* values less than 0.05 were considered to be statistical significant. Data presented is representative of at least three independent experiments. Statistical software, Social Sciences software (SPSS, version 21, Chicago), was used.

**References**

1. Abd El-Rehim DM, Ball G, Pinder SE, Rakha E, Paish C, Robertson JF, et al. High-throughput protein expression analysis using tissue microarray technology of a large well-characterised series identifies biologically distinct classes of breast cancer confirming recent cDNA expression analyses. Int J Cancer. 2005;116(3):340-50.

2. Aleskandarany MA, Agarwal D, Negm OH, Ball G, Elmouna A, Ashankyty I, et al. The prognostic significance of STAT3 in invasive breast cancer: analysis of protein and mRNA expressions in large cohorts. Breast Cancer Res Treat. 2016;156(1):9-20.

3. Aleskandarany MA, Rakha EA, Ahmed MA, Powe DG, Ellis IO, Green AR. Clinicopathologic and molecular significance of phospho-Akt expression in early invasive breast cancer. Breast Cancer Res Treat. 2011;127(2):407-16.

4. Gyorffy B, Lanczky A, Eklund AC, Denkert C, Budczies J, Li Q, et al. An online survival analysis tool to rapidly assess the effect of 22,277 genes on breast cancer prognosis using microarray data of 1,809 patients. Breast Cancer Res Treat. 2010;123(3):725-31.

5. Ciriello G, Gatza ML, Beck AH, Wilkerson MD, Rhie SK, Pastore A, et al. Comprehensive Molecular Portraits of Invasive Lobular Breast Cancer. Cell. 2015;163(2):506-19.

6. Cerami E, Gao J, Dogrusoz U, Gross BE, Sumer SO, Aksoy BA, et al. The cBio cancer genomics portal: an open platform for exploring multidimensional cancer genomics data. Cancer Discov. 2012;2(5):401-4.

7. Gao J, Aksoy BA, Dogrusoz U, Dresdner G, Gross B, Sumer SO, et al. Integrative analysis of complex cancer genomics and clinical profiles using the cBioPortal. Sci Signal. 2013;6(269):pl1.

8. Sarwar M, Semenas J, Miftakhova R, Simoulis A, Robinson B, Gjorloff Wingren A, et al. Targeted suppression of AR-V7 using PIP5K1alpha inhibitor overcomes enzalutamide resistance in prostate cancer cells. Oncotarget. 2016;7(39):63065-81.

9. Semenas J, Hedblom A, Miftakhova RR, Sarwar M, Larsson R, Shcherbina L, et al. The role of PI3K/AKT-related PIP5K1alpha and the discovery of its selective inhibitor for treatment of advanced prostate cancer. Proc Natl Acad Sci U S A. 2014;111(35):E3689-98.

10. Syed Khaja AS, Dizeyi N, Kopparapu PK, Anagnostaki L, Harkonen P, Persson JL. Cyclin A1 modulates the expression of vascular endothelial growth factor and promotes hormone-dependent growth and angiogenesis of breast cancer. PLoS One. 2013;8(8):e72210.
